# The *PAPSS1* gene is a modulator of response to cisplatin by regulating estrogen receptor alpha signaling activity in ovarian cancer cells

**DOI:** 10.1186/s13048-023-01262-7

**Published:** 2023-09-08

**Authors:** Lei Sun, Wei-Xue Ji, Yan Li, Ze-Lian Li, Can-Can Duan, Bai-rong Xia, Lan Xiao

**Affiliations:** 1 Department of Obstetrics & Gynecology, the First Affiliated Hospital, Anhui Medical University, Hefei, 230020 Anhui P. R. China; 2 Department of Pathology and Pathophysiology, School of Medicine, Jianghan University, Wuhan, 430056 Hubei P. R. China; 3 Department of Gynecology Oncology, The First Affiliated Hospital of USTC, Division of Life Sciences and Medicine, University of Science and Technology of China, Hefei, 230031 Anhui P. R. China

**Keywords:** Epithelial ovarian cancer, Sulfation pathways, PAPSS1, Cisplatin resistance, Estradiol, Estrogen receptor alpha

## Abstract

**Background:**

Cancer cells may develop resistance to cisplatin by various mechanisms. Yet, the exact mechanism of cisplatin in ovarian cancer remains unclear. Recent studies have shown that 3’-phospoadenosine 5’-phosphosulfate synthase 1 (PAPSS1) inhibition combined with low-dose cisplatin increases DNA damage. The aim of this study was to determine the value of targeting PAPSS1 as a cisplatin modulator in epithelial ovarian cancer (EOC).

**Results:**

Increased expression of PAPSS1 was observed in both EOC cells and tissues. Also, its higher nuclear expression was distinctly associated with FIGO (The International Federation of Gynecology and Obstetrics) stage, histological subtype, metastasis, and recurrence. Down-regulation of the *PAPSS1* gene increased the cisplatin sensitivity of EOC in vitro and in vivo. Expression of PAPSS1 was negatively correlated with estrogen receptor α (ERα) in EOC. Also, low nuclear PAPSS1 and high nuclear ERα expression in EOC were associated with longer overall survival and progression-free survival in all ovarian cancer and ovarian cancer patients who received platinum-based chemotherapy. PAPSS1 silencing increased the activity of ERα-signaling in EOC cells, thus sensitizing tumors to cisplatin.

**Conclusions:**

These findings characterize a novel interplay between PAPSS1-mediated sulfation and ERα-signaling in EOC cisplatin resistance. PAPSS1 may be exploited as a cisplatin-sensitizing therapeutic target.

**Supplementary Information:**

The online version contains supplementary material available at 10.1186/s13048-023-01262-7.

## Background

Ovarian cancer has the highest mortality rate among all gynecologic malignancies [1], with a 5-year survival rate of < 40% [2, 3]. Epithelial ovarian cancer (EOC) is the most common subtype, accounting for 90% of all ovarian cancers. Patients with EOC are usually given chemotherapy, and the most popular drugs include platinum drugs, cisplatin, and carboplatin [4, 5]. Yet, while most EOC tumors initially respond well to platinum-based therapy, about 75% of patients experience disease relapse due to high therapeutic resistance [6–8]. In order to improve the prognosis of EOC patients, it is of urgent importance to elucidate the underlying mechanisms that promote platinum resistance in EOC.

Estrogen can induce the growth of cancer cells through multiple pathways, including estrogen receptor (ER)-mediated pathways [9]. Sulfation is the main pathway for estrogen metabolism [10]. Sulfation of active estradiol (E_2_) forms inactive estradiol sulfate, which can be reactivated following desulfation by estrogen sulfatase. 3'-phosphoadenosine 5'-phosphosulfate (PAPS) synthase (PAPSS) catalyzes the biosynthesis of PAPS, which serves as the universal sulfonate donor compound for all sulfotransferase reactions [11]. In humans, PAPSS exists in two isoforms: PAPSS1 (3’-phospoadenosine 5’-phosphosulfate synthase 1) and PAPSS2(3’-phospoadenosine 5’-phosphosulfate synthase 2) [12, 13]. PAPSS1 is localized to the nucleus, while PAPSS2 is found in the cytoplasm [14, 15]. PAPSS1 sequentially synthesizes the biologically active sulfate form, the substrate for cell sulfonation reactions.

Sulfonation has been largely overlooked in the context of oncology. Recent evidence has suggested that the sulfonation pathway may contribute to carcinogenesis and patient survival. For example, Xu et al*.* demonstrated that the overexpression of SULT1E1 and PAPSS1 can block estrogen-stimulated cell proliferation in MCF-7 breast cancer cells [16]. Also, *PAPSS1* has been suggested as a candidate HCC-susceptibility gene and correlated with poor survival in patients with familial or early onset hepatocellular carcinoma (HCC) [17, 18]. Alterations in the *PAPSS2* have been associated with bone development diseases, hepatocellular carcinoma, and estrogenic hormone disorder [19]. A lower expression of PAPSS2 has been correlated with worse survival in patients with colon cancer [20]. Moreover, recent studies suggested that silencing of PAPSS1 can enhance cisplatin activity in non-small cell lung cancer; also, PAPSS1 expression was negatively correlated with survival rate in patients receiving platinum-based chemotherapy [21, 22]. However, studies on PAPSS association with cancer are still in their infancy, especially studies assessing PAPSS enzymes with platinum-based chemotherapy. Also, the exact mechanisms of action remain unclear.

EOC is characterized by DNA repair defects [23], especially the homologous recombination repair (HRR) deficiency. HRR-deficient tumors frequently originate from hormone-enriched tissues, such as breast and ovarian tissue [24, 25]. It has also been found that estrogen increases genome instability affecting HRR in estrogen receptor-positive (ERα +) EOC cells [26]. Platinum functions through exacerbating DNA damage; these drugs are considered DNA damage-inducing drugs, which might disrupt the DNA repair pathway, increase reactive oxygen species, and ultimately lead to DNA damage-dependent apoptosis/cell death [27, 28]. The participation of estrogen and ERs in the development of chemoresistance to cisplatin is observed in multiple cancer types, including breast cancer, non-small cell lung cancer and ovarian cancer [29–31]. Contrary, some studies showed that estrogen decreases resistance to cisplatin in vitro [32, 33]. Therefore, a better understanding of the contribution of estrogen and ERs to the emergence of resistance to cisplatin in EOC will enable us to identify targets in this pathway in order to restore sensitivity to cisplatin chemotherapy.

In the current study, we determined the therapeutic value of targeting PAPSS1 as a cisplatin modulator in vitro and in vivo by testing the effects of *PAPSS1* gene knockdown on cisplatin activity in EOC cells. To understand the interaction of PAPSS1 and ERα on a molecular level, we investigated the expression and their correlation in vitro.

## Results

### PAPSS1 expression in EOC tissues and cell lines and its association with cisplatin-resistance

**Fig. 1:**
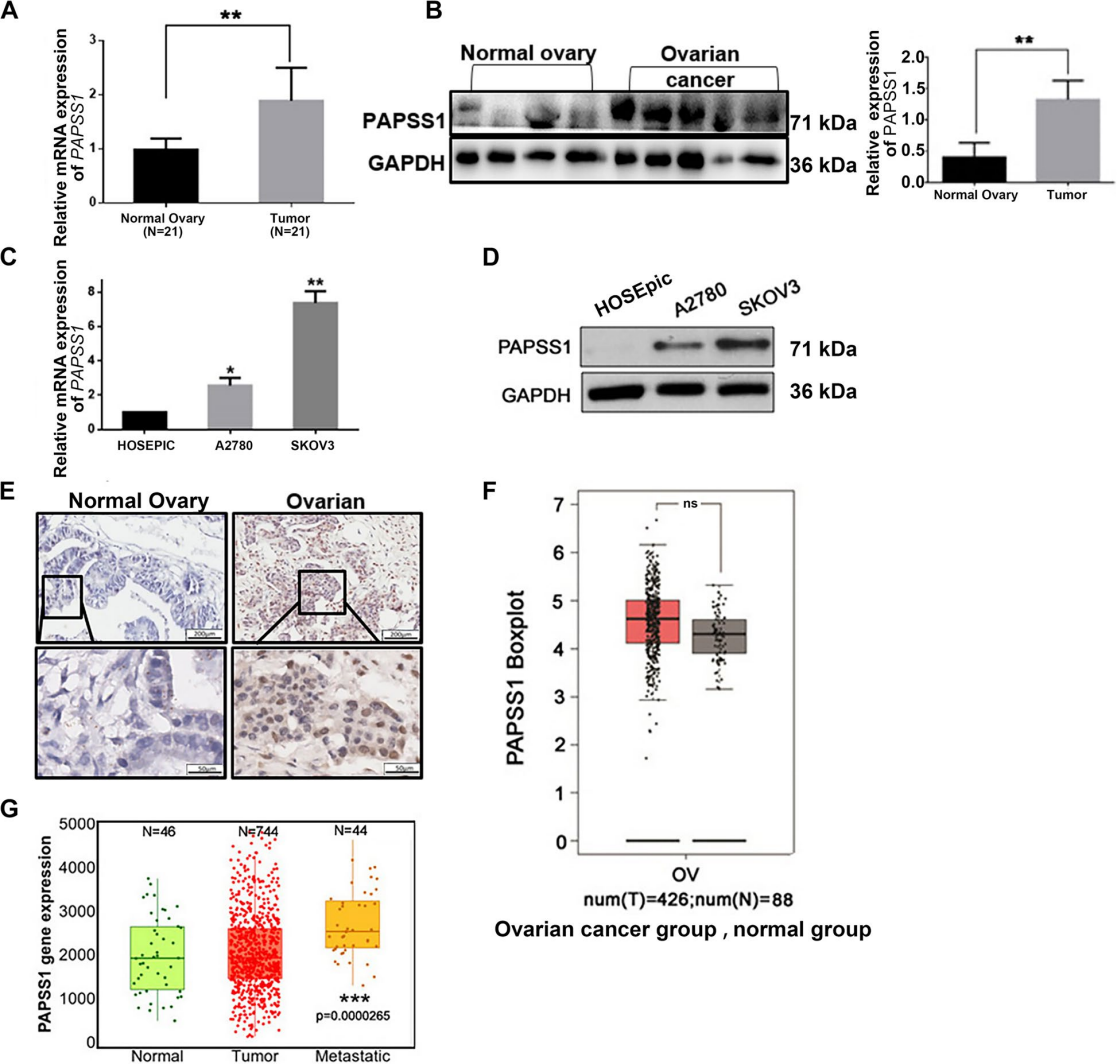
PAPSS1 expression in EOC tissues and cell lines and role in cisplatin resistance. **A**, **B** Expression of *PAPSS1* mRNA (A) and protein (B) in the normal ovarian epithelium and ovarian cancer tissues (21 pairs of EOC and epithelial normal ovary tissues) analyzed by qRT-PCR (A) and Western blot (B). **C**, **D** The expression of *PAPSS1* mRNA (C) and protein (D) in EOC and normal ovarian cell lines (HOSEpic cells, cisplatin-sensitive A2780 cells, and cisplatin-resistant SKOV3 cells) by qRT-PCR (C) and Western blot (D). **E** Representative images and its regional magnification of PAPSS1 IHC in ovarian cancer and normal ovarian epithelium tissues: high expression of PAPSS1 is seen in ovarian cancer tissues; low expression of PAPSS1 is seen in the nuclear of normal ovarian epithelium tissues. Scale bar: 200 µm and 50 µm. **F** Relative expression of PAPSS1 in ovarian cancer and epithelial normal ovary tissues by boxplot graph identified based on TCGA datasets (ovarian cancer *n* = 426 and normal ovarian tissues *n* = 88). Image data derived from TCGA Ovarian Cancer Database information, where N indicates *P* > 0.05 (not a statistically significant difference). The red is tumor tissue and grey is normal tissue. **G** PAPSS1 transcript expression is upregulated in metastatic tissues relative to the primary tumor and normal tissue. Data shown represent the mean ± SD. ^N^*P* > 0.05; *P < 0.05. **P < 0.01, ***P < 0.001

Firstly, the expression of PAPSS1 was examined in 21 pairs of EOC and epithelial normal ovary tissues by q-PCR and Western blot. The mRNA expression in EOC tissues was 1.95 times higher than in normal ovarian epithelium (*P* < 0.05, Fig. 1A). Besides, the protein level significantly increased in EOC tissues compared to the normal ovarian epithelium(*P* < 0.05, Fig. 1B).

Next, we assessed the mRNA and protein expression of PAPSS1 in normal ovarian HOSEpic cells, cisplatin-sensitive A2780 cells, and cisplatin-resistant SKOV3 cells. As shown in Fig. 1C, the mRNA level of *PAPSS1* in cisplatin resistance SKOV3 cells was 7.62 times higher than HOSEpic cells and 2.64 times higher than cisplatin-sensitive A2780 cells. Furthermore, the PAPSS1 protein was highly expressed in SKOV3 cells compared with HOSEpic and A2780 cells (*P* < 0.05, Fig. 1D).

The increased expression of PAPSS1 in human ovarian cancer was further validated by IHC analysis of an ovarian cancer tissue array, including 131 ovarian cancer tumors. The results indicated that PAPSS1, predominantly observed in the nucleus of cells and EOC tissues, showed stronger staining than normal ovarian epithelium tissues (Fig. 1E). Also, according to TCGA and GTEx mRNA data, *PAPSS1* showed a higher expression in ovarian cancer tissues(Fig. 1F). Finally, PAPSS1 showed increased PAPSS1 expression in tumor and metastatic tissues of the ovary compared to normal tissues (Fig. 1G). These data highlight the profound role of PAPSS1 in EOC treatment. Also, these data suggest that PAPSS1 might be involved in the cisplatin resistance of EOC.

### PAPSS1 regulates EOC proliferation and sensitivity to cisplatin in vitro

**Fig. 2:**
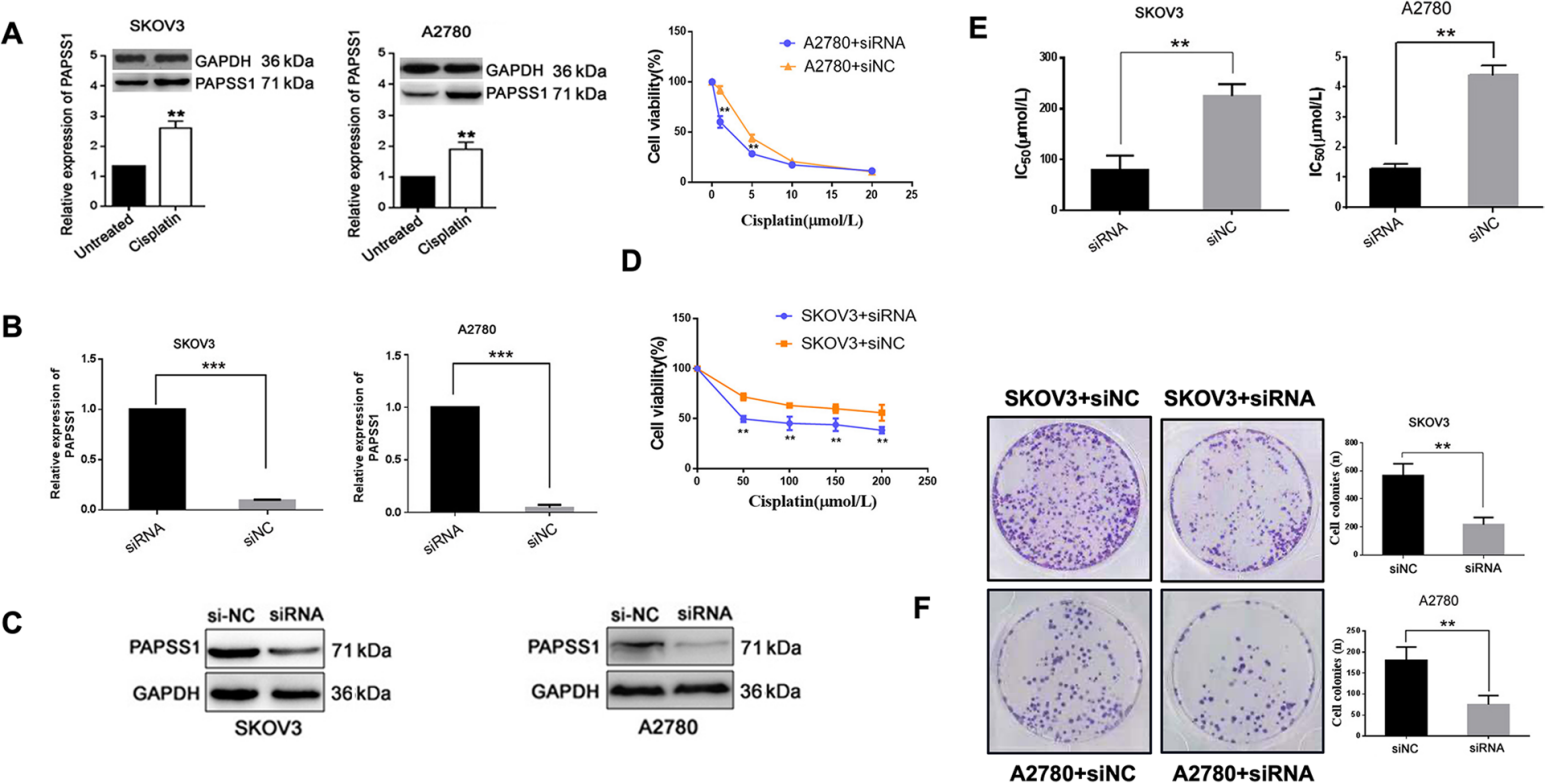
PAPSS1 regulates EOC cell proliferation and sensitivity to cisplatin in vitro. **A** Western blots show the relative PAPSS1 protein levels in untreated and cisplatin-sensitive A2780 cells and cisplatin-resistant SKOV3 cells. Densitometric analysis of the levels of PAPSS1 protein is shown at the bottom in A. The black column represents untreated cells, and the white column represents cisplatin treatment cells. **B**, **C** The transfection efficiency of PAPSS1 siRNA determined by qRT-PCR and Western blots. **D**, **E** The proliferation and the cisplatin IC_50_ of PAPSS1 silenced cells determined by CCK-8 assay. **F** Cells were treated with the indicated doses of cisplatin for 24 h, and colony formation assays were conducted to determine the cell survival ability for siPAPSS1 transfected EOC cells. Data shown represent the mean ± SD. *** *P* < 0.001,** *P* < 0.01

The basal level of PAPSS1 was higher in SKOV3 than in A2780 cells, and cisplatin led to an up-regulation of PAPSS1 in two cells (Fig. 2A). To further investigate the functional role of PAPSS1 in EOC, we examined the effects of PAPSS1 silencing in A2780 and SKOV3 cell lines using PAPSS1 siRNA (siPAPSS1) and the negative control (si-NC). siPAPSS1 strongly suppressed *PAPSS1* mRNA in SKOV3 or A2780 cells; mRNA level was reduced by 84.8% in SKOV3 and by 91% in A2780 compred to si-NC gorups (all *P* < 0.001) (Fig. 2B). Furthermore, the PAPSS1 protein level decreased following 48 h transfections (all *P* < 0.05, Fig. 2C). Moreover, the cell viability of A2780 transfected with siPAPSS1 and SKOV3 transfected with siPAPSS1 cells was significantly decreased compared with A2780 and SKOV3 cells transfected with si-NC (all *P* < 0.05, Fig. 2D, E). Moreover, colony formation assay indicated that PAPSS1 knockdown reduced the clonogenicity of A2780 and SKOV3 cells compared to cells transfected with the si-NC (all *P* < 0.05, Fig. 2F). These results suggested that PAPSS1 plays an important role in regulating the response to cisplatin in two cells.

### PAPSS1 regulates EOC cell apoptosis and cell cycle

**Fig. 3:**
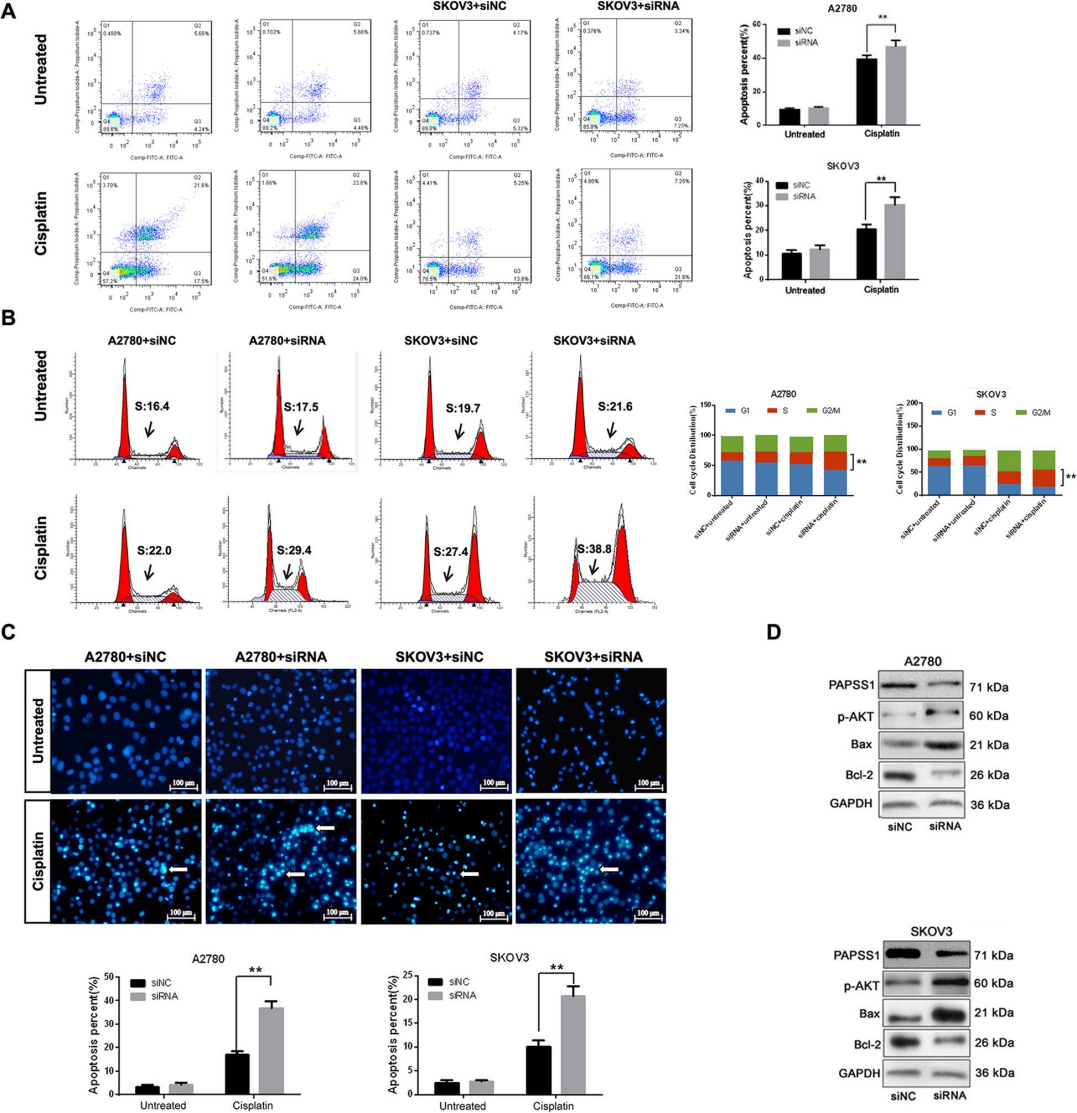
PAPSS1 regulates EOC cell apoptosis and cell cycle. **A**, **B** A2780 and SKOV3 cells were treated with low doses of cisplatin (1 or 10 μM) for 24 h. Flow cytometry was performed to determine the apoptosis and cell cycle of PAPSS1 silenced cells (the black arrow points to the S phase block in the cell cycle of EOC cells). Densitometric analysis of the apoptotic rate and cell cycle are shown in the right side in A and B. **C** A2780 and SKOV3 cells were treated with low doses of cisplatin (1 or 10 μM) for 24 h, respectively. Cells were stained with Hoechst 33342. Highly condensed or fragmented nuclei represent apoptotic cells. Intact nuclei represent viable cells. The white arrows indicate early-stage apoptotic nuclei. Densitometric analysis of the apoptotic cell rate is shown at the bottom in C. Scale bar: 100 µm. **D** Apoptosis-related protein Bax, Bcl-2 and p-AKT levels were determined by Western blots. Images were visualized using the in Cell Analyzer 2200. Data shown represent the mean ± SD. ** *P* < 0.01

To confirm the apoptotic resistance effect of PAPSS1, siRNA-transfected A2780 and SKOV3 cells were treated with IC_50_ of cisplatin. The flow cytometry results demonstrated that the apoptotic rate was significantly increased in cisplatin-treated A2780 and SKOV3 cells following PAPSS1 silencing (Fig. 3A). On the other hand, PAPSS1 knockdown A2780 and SKOV3 cells treated with cisplatin presented more apoptotic features of nuclei stained with Hoechst 33342, including prominent nucleus shrinking with increased density (Fig. 3B). Also, asignificant increase at the S phase was observed on cisplatin-treated A2780 and cisplatin-treated SKOV3 cells transfected with siPAPSS1 compared with A2780 and SKOV3 cells transfected with si-NC (*P* < 0.01) (Fig. 3C). In addition, decreased ratio of Bax/Bcl-2 was observed in cisplatin-treated A2780 and cisplatin-treated SKOV3 cells transfected with siPAPSS1 (Fig. 3D) and PAPSS1 inhibition did affect the phosphorylation status of Akt. These results demonstrate that PAPSS1 impacts the sensitivity to cisplatin in EOC cells through apoptosis and cell cycle regulation.

### PAPSS1 is involved in the DNA damage process in EOC cells sensitive to cisplatin

**Fig. 4:**
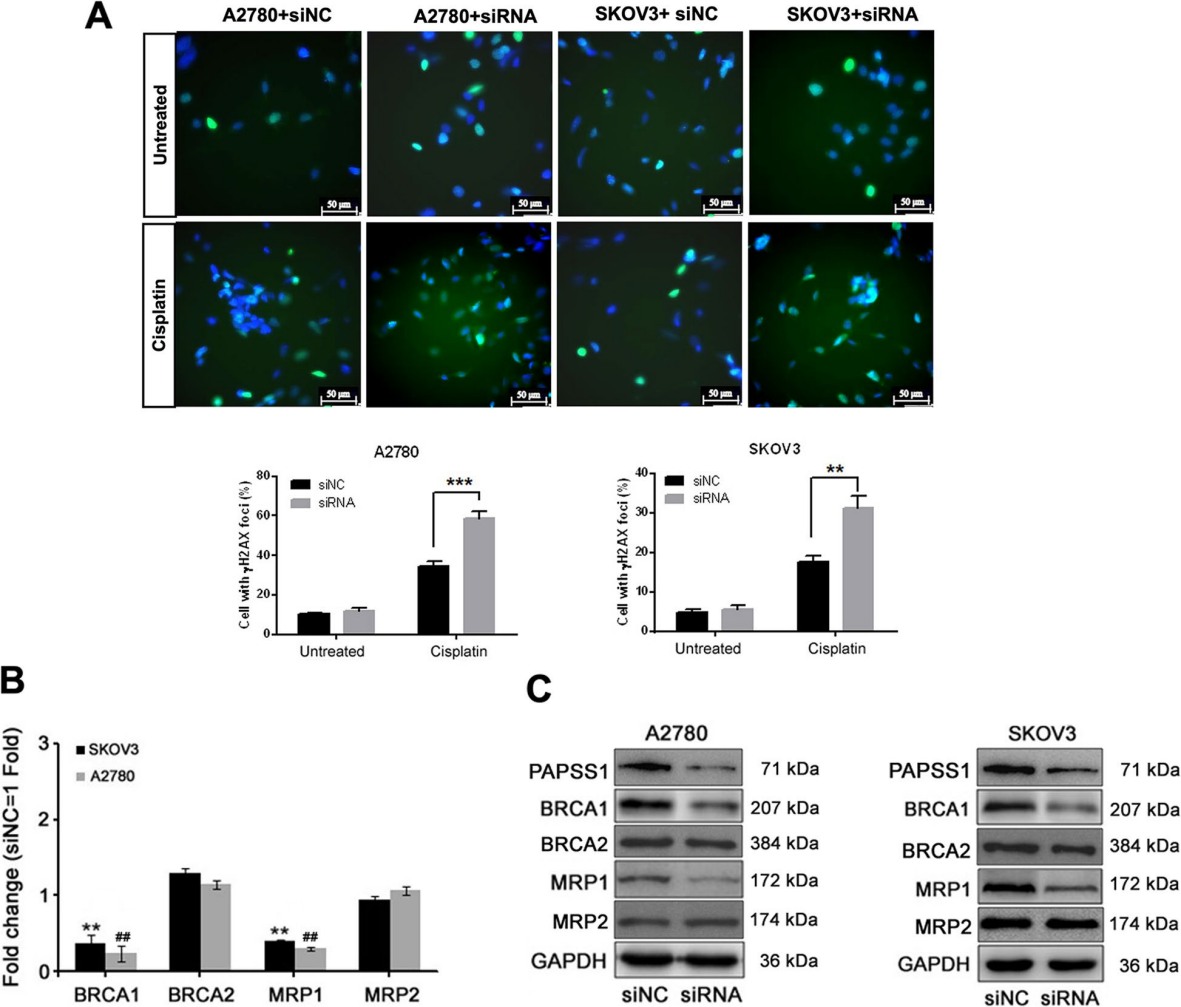
PAPSS1 regulates EOC cell DNA damage. **A** For immunofluorescent staining, A2780 and SKOV3 cells were treated with low doses of cisplatin (1 or 10 μM) for 24 h, respectively. Cells stained with anti-γH2AX antibody (green) and counter-stained with DAPI (blue). The percentage of cells with more than one γH2AX-positive puncta are quantified and plotted at the bottom in A. Scale bar: 50 µm. The data shown represent the mean ± SD. ** *P* < 0.01,*** *P* < 0.001. **B**, **C** The expression of genes related to cell DNA repair (*BRCA1*, *BRCA2*) and drug transporters (*MRP1*, *MRP2*) in two siRNA cells compared to two si-NC cells examined by qRT-PCR. Western blot (**C**). The gene expression in fold-change was calculated by the delta-delta Ct method. The data shown represent the mean ± SD. Statistically significant (*P* < 0.01) changes in SKOV3 and A2780 cells are indicated by symbols ** and ^##^, respectively

Altered expression of genes associated with DNA damage-dependent apoptotic response and drug transporters influence chemotherapeutic response. The γH2AX, a phosphorylated form of histone H2AX that functions as a sensitive marker for double-strand breaks, is triggered inthe very early phase of DNA damage and can form damage-induced foci in the chromatin regions of damaged DNA [34]. Thus, we next investigated the effect of PAPSS1 on DNA damage in EOC cells. Figure 4A shows the representative images of γH2AX foci in si-NC and siPAPSS1-transfected cells treated with low doses of cisplatin. Also, immunofluorescence staining showed significantly more cells with γH2AX-positive puncta in PAPSS1 silencing EOC cells **(**Fig. 4A).

Next, we performed qRT-PCR and Western blot to confirm further that homologous combination repair modules (*BRCA1, BRCA2*) and drug transporters (*MRP1*, *MRP2*) mRNA and protein levels to understand their role in increased sensitivity of cisplatin in PAPSS1 silenced EOC cells. The knockdown of *PAPSS1* resulted in the decreased *BRCA1* mRNA and protein in the cisplatin-treated A2780 and SKOV3 cells (Fig. 4B, C). Conversely, two cells transfected with siPAPSS1 showed lower expression of MRP1 than two cells transfected with the si-NC (Fig. 4B, C). These results suggest that PAPSS1 reduces the amount of DNA damage caused by cisplatin, blocks DNA repair, and regulates MRP efflux in the cisplatin resistance to EOC cells.

### Silencing of PAPSS1 improves in vivo sensitivity to cisplatin

**Fig. 5:**
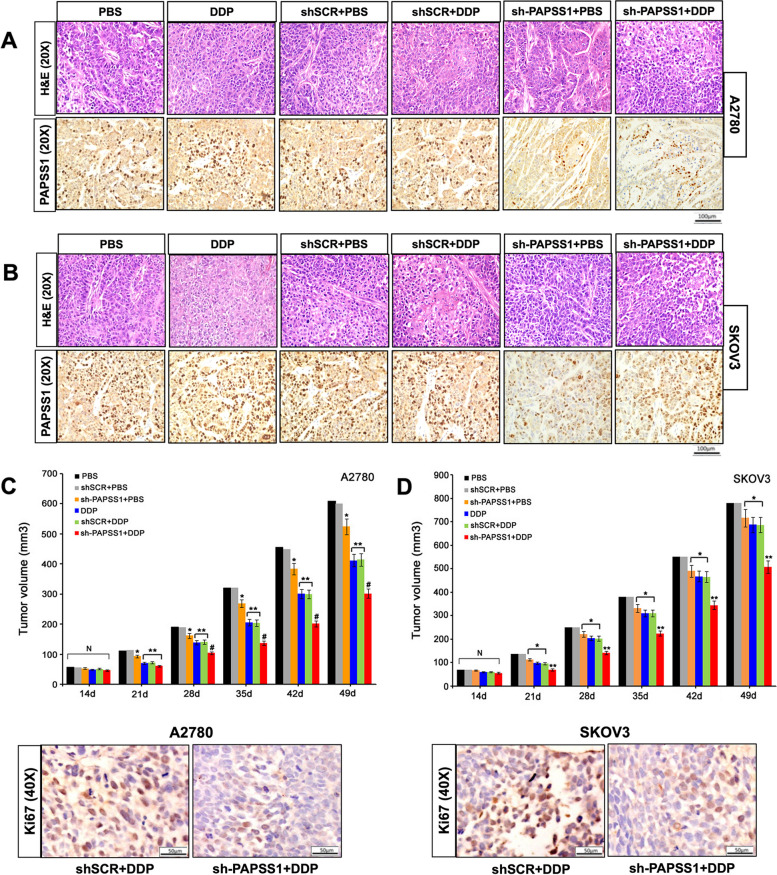
Silencing of PAPSS1 improves in vivo sensitivity to cisplatin. **A**, **B** Representative H&E and IHC staining of PAPSS1 in different groups. Scale bar: 100 µm. **C**,** D** The volume and ki67 expression of the ovarian tumor xenograft derived from sh-PAPSS1 transfected A2780 and SKOV3 cells were significantly lower than those derived from shSCR transfected A2780 and SKOV3 cells. Scale bar: 50 µm. Data are shown as mean ± SE. N vs. PBS and shSCR + PBS, *P* > 0.05; * vs. PBS and shSCR + PBS, *P *< 0.05; ** vs. PBS and shSCR + PBS, *P* < 0.01; ^#^ vs. PBS and shSCR + PBS, *P* < 0.001

To further investigate the effect of PAPSS1 on tumor growth, nude mice were inoculated subcutaneously with A2780 and SKOV3 cells transfected with sh-PAPSS1 or shSCR, followed by cisplatin treatment (0.3 or 3 mg/kg) used for both ovarian tumor xenografts derived from A2780 and SKOV3 cells. The volume of ovarian tumor xenografts was analyzed 49 days post-inoculation. IHC staining showed the expression of PAPSS1 in the tumor xenografts derived from A2780 and SKOV3 cells increased after DDP administration compared with the PBS and shSCR + PBS groups. Besides, following DDP treatment, there was a decreased PAPSS1 staining in the ovarian tumor xenografts derived from sh-PAPSS1 transfected A2780 and SKOV3 cells compared with the shSCR transfected A2780 and SKOV3 cells, suggesting that enhanced efficacy of cisplatin treatment in cells with suppressed PAPSS1 expression(Fig. 5A, B). Ovarian tumor xenografts with PAPSS1-targeting shRNAs was determined to be effective at slowing the rate of tumor growth in animals, and PAPSS1 suppression/cisplatin combination was superior to cisplatin in the inhibition of tumor growth. (Fig. 5C, D). Furthermore, as determined by immunocytochemistry, decreased Ki-67 levels were detected in PAPSS1 silenced tumors compared with the shSCR groups. These results revealed that suppression of PAPSS1 expression inhibits EOC progression by enhancing the in vivo sensitivity to cisplatin.

### Low PAPSS1 and high ERα expression are associated with improved OS, PFS and cisplatin sensitivity in patients with ovarian cancer

**Fig. 6:**
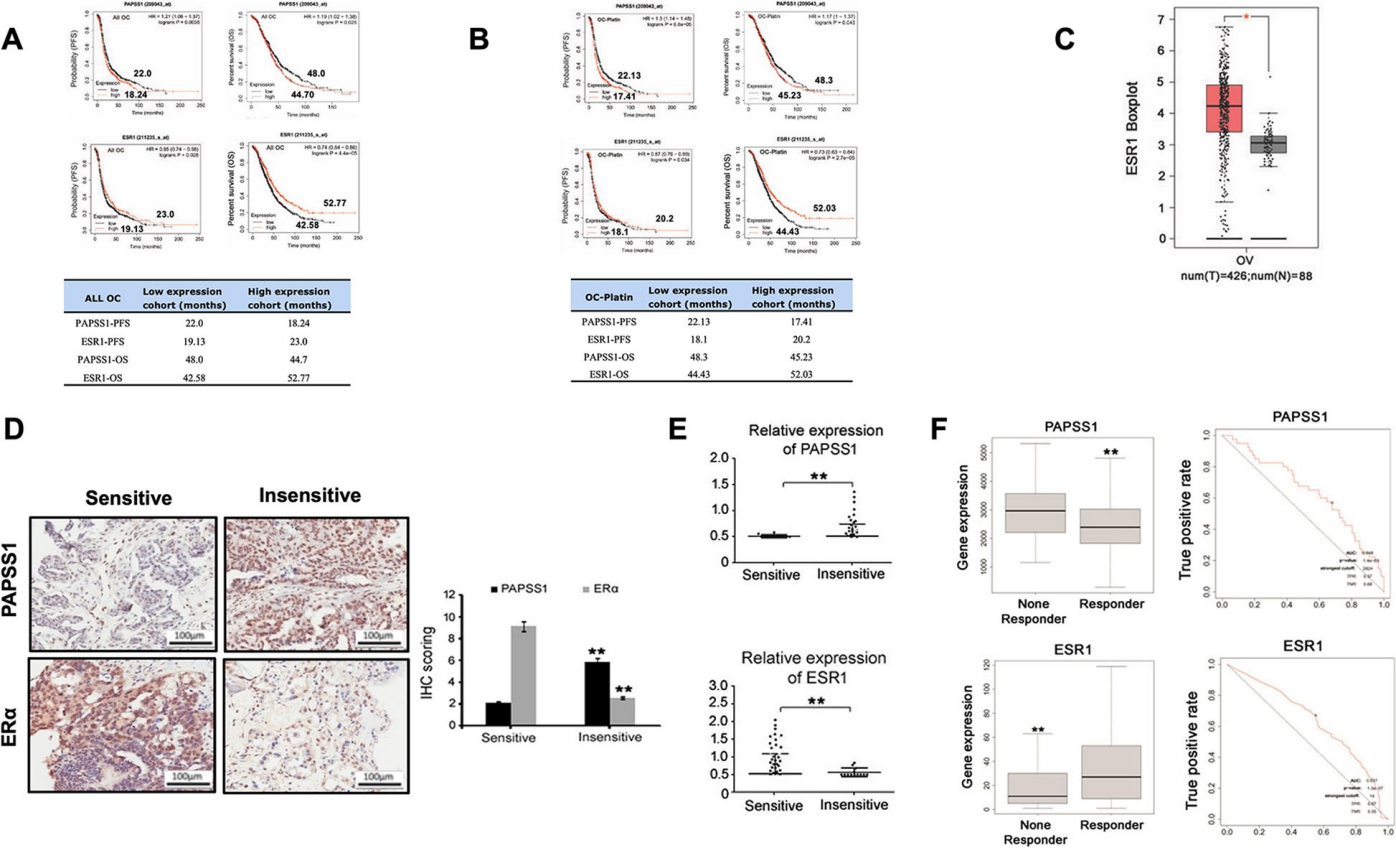
Low PAPSS1 and high ERα associated with improved OS, PFS and cisplatin-sensitivity in ovarian cancer patients. **A** Kaplan–Meier plots of overall survival and PFS in OC patients stratified according to their PAPSS1 (*P* = 0.025, log-rank test) and ESR1 (*P* = 0.00045, log-rank test) status. The side panel on the right in Fig. 6A illustrates a median survival time for PFS and OS comparison of ESR1 and PAPSS1 expression in ovarian cancer patients. **B** Kaplan–Meier plots of overall survival and PFS in OC patients for which platin-based chemotherapy stratified according to their PAPSS1 (*P* = 0.045, log-rank test) and ESR1 (*P* = 0.000027, log-rank test) status. The panel at the bottom in Fig. 6B illustrates a median survival time for PFS and OS comparison of ESR1 and PAPSS1 expression in platin-based chemotherapy ovarian cancer patients. **C** Data from TCGA showed that ESR1 has a high expression level in ovarian cancer tissues (ovarian cancer *n* = 426 and normal ovarian tissues *n* = 88) (*P < 0.05). The red is tumor tissue and grey is normal tissue. **D** IHC staining shows lower ERα and higher PAPSS1 expression in platinum-resistant OC specimens than in non-resistant specimens. The IHS of PAPSS1 and ESR1 in the OC tissue assay are summarized as the normalized means of scores ± standard error (SE) on the right side in D (**P < 0.01). Scale bar: 100 µm. **E** Relatively expression of *PAPSS1* and *ESR1* mRNA in OC tumor platinum resistance and platinum-sensitive specimens by beeswarm plot (**P < 0.01). **F** ROC curves and boxplots of PAPSS1 and ESR1 in ovarian cancer patients undergoing platinum treatment for relapse-free survival time of 6 months. Area under curve (AUC); TNR true negative rate (TNR); true positive rate (TPR) (http://www.rocplot.org/, accessed on October 2021)

Kaplan–Meier survival analysis revealed a longer progression-free survival (PFS) and overall survival (OS) in ovarian cancer patients with low PAPSS1 expression and high ESR1 expression than those with low ESR1 and high PAPSS1(Fig. 6A). Besides, low PAPSS1 and high ESR1 were associated with improved PFS and OS in platin-based chemotherapy ovarian cancer patients (Fig. 6B). Our study showed an inverse correlation of the gene expression between ESR1 and PAPSS1 in Kaplan–Meier plotter platform of ovarian cancer and platin-based chemotherapy ovarian cancer patients. ESR1 expression in ovarian cancer and epithelial normal ovary tissues is presented as a boxplot graph identified based on open-source TCGA and GTEx mRNA data using the GEPIA online tool (Fig. 6C).

To further determine whether PAPSS1 and ESR1 are differentially expressed in platinum-resistant and platinum-sensitive EOC cases, EOC clinical tissues were subdivided into two platinum-resistant and platinum-sensitive groups. As shown in Fig. 6D, IHC staining showed a lower ERα and a higher PAPSS1 expression in platinum-resistant specimens than in platinum-sensitive specimens. In addition, the summarized IHS (immunohistochemical scoring) revealed high PAPSS1 and low ERα scores in the platinum-resistant tissues of EOC patients. However, relative *PAPSS1* mRNA levels were higher in the platinum-resistant than in the platinum-sensitive group (1.06 ± 0.34 vs. 0.43 ± 0.11; *P* < 0.01). In comparison, ESR1 was strongly expressed in platinum-sensitive cases compared to the platinum-resistant cases (2.07 ± 0.28 vs. 0.54 ± 0.21; *P *< 0.01) (Fig. 6E). We also performed follow-up analyses by considering patients who had an event within 3 years of follow-up. Follow-up analyses showed that median overall survival was significantly longer in platinum-sensitive cases as compared with platinum-resistant cases (22.8 months vs. 8.1 months).

To further analyze the potential of PAPSS1 and ESR1 as the predictive marker for therapy effectiveness in ovarian cancer, a receiver operating characteristic (ROC) analysis was performed to link gene expression to respond to therapy. We compared *PAPSS1* and *ESR1* gene expression to platinum treatment in EOC specimens responders and non-responders. Both *PAPSS1* and *ESR1* gene expression are relatively sensitive markers for predicting the effect of platinum treatment on patients with ovarian cancer. In addition, an inverse correlation of the gene expression between *ESR1* and *PAPSS1* was observed in ROC plotter datasets of ovarian cancer (Fig. 6F). The correlation of PAPSS1 expression with clinicopathological characteristics in tissues of EOC patients is further summarized in Table 1. We found that nuclear PAPSS1 overexpression in EOC was significantly correlated with the FIGO stage, histological subtype, platinum resistance, metastasis, and recurrence. Thus, our findings indicate that PAPSS1 and ERα expression correlates with the prognosis and response to cisplatin-based chemotherapy in EOC patients. Combined with PAPSS1 and ESR1 may efficiently discriminate cisplatin-resistant and sensitive EOC.

**Table 1:** Association of PAPSS1 expression with clinicopathological factors in ovarian carcinoma patients

Features		n	PAPSS1		χ2	P
			**Positive**	**Negative**		
Mean age	> 50	79	49	30	0.715	0.398
Ascites	≤ 50	52	36	16		
	Positive	72	43	29	1.871	0.171
	Negative	59	37	22		
FIGO stage	I-II	68	35	33	11.168	0.001
	III-IV	63	50	13		
CA125 (U/ml)	≥ 200	79	48	31	1.487	0.223
	< 200	52	37	15		
Pathological Subtype	Serous	92	71	21	20.481	0.000
	Non serous	39	15	25		
Tumor grade	G1	23	11	12	7.236	0.053
	G2	70	43	27		
	G3	38	22	16		
Tumor	< 10	89	58	31	0.010	0.921
Size	≥ 10	42	27	15		
Recurrent	Yes	82	59	23	4.804	0.028
(3 Years)	No	49	26	23		
Metastasis	Yes	84	57	27	6.364	0.041
	No	47	23	24		
CRS	Yes	84	55	29	0.036	0.850
	No	47	30	17		
Platinum resistance	Present	35	27	8	4.395	0.040
	Absent	96	50	46		

### Downregulation of PAPSS1 increases ERα and E_2_ expression, confirming their genetic interaction, and mediated cisplatin resistance in EOC

**Fig. 7:**
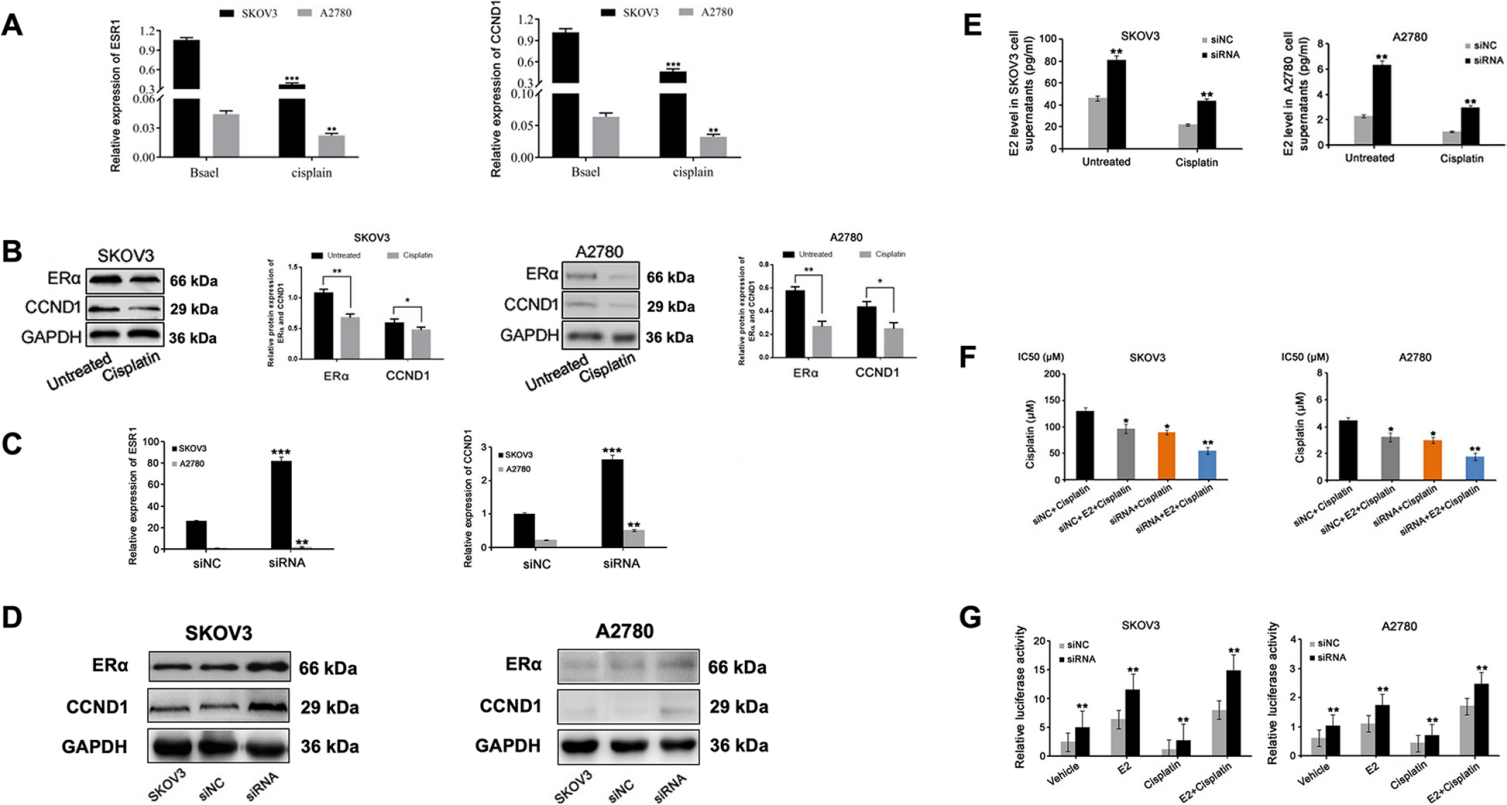
Downregulation of PAPSS1 increases ERα and E_2_ expression, mediating cisplatin resistance in EOC. **A**, **B** mRNA and Western blot expression levels of ESR1 and CCND1 in A2780 and SKOV3 cells with or without cisplatin (2 or 50 μM); **P < 0.01, ***P < 0.001 vs. untreated two cells. **C**, **D** mRNA and Western blot expression levels of ESR1 and CCND1 in siPAPSS1 and si-NC transfected A2780 and SKOV3 cells (**P < 0.01, ***P < 0.001 vs si-NC two cells). **E** The E_2_ level in cell supernatants in two siRNA cells compared to two si-NC cells with or without cisplatin (2 or 50 μM) examined by ELISA 24 h later. **F** After 24 h of transfection, cells were treated with cisplatin + E_2_ (1 nM) for 24 h. CCK-8 assay revealed the cisplatin IC_50_ in two siRNA cells as compared to two si-NC cells. **G** A2780 and SKOV3 cells were plated in 6-well plates 24 h before transfection. Two cells were transfected with ptk-ERE-luc and pRLCMV as an internal control. After 24 h of serum-free starvation, A2780 and SKOV3 cells were treated with vehicle, E2 (1 nM), cisplatin (2 or 50 μM), E2 (1 nM) + cisplatin (2 or 50 μM) for 24 h. Cell lysates were assayed for luciferase activity. Luciferase activity was normalized against Renilla luciferase using the pRLCMV control vector. The data shown represent the mean ± SD. *** *P* < 0.001. ** *P* < 0.01. *P < 0.05

Given that estrogen and estrogen receptor (ER) mediated cisplatin chemoresistance in cancer, we aimed to examine if this would be reflected in the level of related gene expressions. A2780 and SKOV3 cells were treated with 2 μM or 50 μM of cisplatin for 24 h, respectively. qRT-PCR and Western blot were then used to confirm the *ERα* and *CCND1* mRNA (Fig. 7A) and protein (Fig. 7B) levels in the two cells. The basal level of ERα and CCND1 was higher in SKOV3 than in A2780 cells, and cisplatin led to an apparent down-regulation of ERα and CCND1 in two cells. However, the knockdown of PAPSS1 increased the expression of ERα and CCND1 in SKOV3 and A2780 cells (Fig. 7C, D). At 24 h post-transfection, A2780 and SKOV3 cells in the siPAPSS1 group and si-NC group were cultured in media containing 2 μM or 50μΜ cisplatin for 24 h. The results showed that E_2_ levels in the siPAPSS1 group were significantly increased compared to those in the si-NC group in two cells (Fig. 7E).

Next, we tested the sensitivity of A2780 and SKOV3 cells to cisplatin and the effect of PAPSS1 and estrogenon cisplatin-induced cytotoxicity using the CCK-8 assay. Exposure to E_2_ increased the sensitivity to cisplatin in two si-NC cells. Further titrations revealed that co-treated with E_2_ further increased the sensitivity of cisplatin in two siRNA cells compared to two si-NC cells (Fig. 7F). In addition, we also examined the effects of cisplatin on the transcriptional activation of ERE via ERa. We transfected the ER-responsive receptor plasmid, ptk-ERE-luc, into SKOV3 and A2780 cells and performed a luciferase assay. As expected, two cells showed increased luciferase activity after stimulation with E_2_. Conversely, cisplatin caused a decrease in luciferase activity compared with vehicle-treated EOC cells. In addition, we found that PAPSS1 silencing of SKOV3 and A2780 cells showed more significant luciferase induction than that from si-NC-transfected SKOV3 and A2780 cells in both groups (Fig. 7G). All these results indicated that PAPSS1 regulates the expression of estrogen α signaling and affects the sensitivity of cisplatin treatment in EOC.

## Discussion

To the best of our knolwdge, this is the first study that revealed a relationship between PAPSS1-mediated sulfation and ERα signaling in cisplatin resistance and that PAPSS1 may have an important role in regulating cisplatin resistance in EOC. There have been significant research interests in the clinical impacts of hormone receptors on ovarian cancer concerning both patients’ survival and drug responsiveness. However, the effect of estrogen signaling on EOC platinum-resistance remains controversial. Some studies found that ER activation by estrogen and cisplatin can induce platinum-resistance by increasing the expression of an anti-apoptotic protein [35, 36]. On the other hand, some other studies demonstrated that estrogen signaling enhances the sensitivity of ovarian cancer cells to chemotherapy agents [37, 38]. Sulfonation, the main pathway for estrogen metabolism, is commonly associated with the metabolism of xenobiotics that inactivate drugs by increasing their water solubility and biological activity [39]. This modification is also partially responsible for drug resistance to chemotherapy in cancer treatments [40]. Given the biological role of PAPSS1, which synthesizes the biologically active form of sulfate, one can speculate on the role of sulfur metabolism and homeostasis in cancer cells when they are first exposed to cytotoxic agents [41]. Thus, studies on PAPSS1 and drug resistance are still in the early stage and thus, more research is required.

In this study, we found that PAPSS1 was highly expressed in ovarian cancer compared to normal tissue and was also upregulated in ovarian cancer cisplatin-sensitivity or resistance cells (A2780 and SKOV3) than in normal ovarian cells (HOSEpic). CCK-8 assay, colony formation assay, apoptosis and cell cycle analysis further revealed that the knockdown of PAPSS1 inhibits cell proliferation and survival, promotes apoptosis, and increases the number of cells in replicating S phase. Next, we found that the knockdown of PAPSS1 may enhance DNA damage in the presence of low doses of cisplatin and down-regulate HRR DNA repair protein BRCA1. Previous studies have shown that drug transporters, such as multidrug resistance-associated protein 1 (MRP1), influence the sensitivity of cancer cells to chemotherapy [42, 43]. In this study, we found a decreased expression of MRP1 in PAPSS1-silenced EOC cells. Furthermore, we demonstrated that suppression of PAPSS1 expression inhibits tumor progression by enhancing the in vivo sensitivity of A2780 and SKOV3 cells to cisplatin. Similarly, Leung et al*.* found that PAPSS1 knockdown sensitizes non-small cell lung cancer ( (NSCLC) cells to cisplatin in vivo [21]. Altogether, these data suggest that the effects achieved when cisplatin is combined with PAPSS1 silencing are highly synergistic in EOC cells.

The present study further investigated the clinical value and significance of PAPSS1. The results of the Spearman chi-square test (Table 1) indicated that the high nucleus PAPSS1 was positively associated with the FIGO stage, histological subtype, platinum resistance, metastasis and recurrence in patients with ovarian cancer. Based on the present findings, we propose that PAPSS1 is a relevant oncology target in ovarian cancers and provides a novel strategy for ovarian cancer treatment.

Studies show that the hormone receptor, especially ER, is significantly associated with improved OS in patients with EOC [44]. In postmenopausal patients with advanced-stage HGSOC, a poorer survival outcome was associated with low functional ER pathway activity [45]. Some studies revealed that ER-positive breast cancers receiving anti-hormone and/or chemotherapy might lose their ER expression, which in turn leads to the disease's evolution to higher aggressiveness and drug resistance [46]. At the same time, a recent study demonstrated that depleting ERα in EOC cells up-regulates HRR activity and HRR gene expression [47]. This study found that both PAPSS1 and ERα are prognostic factors in EOC and are associated with platinum sensitivity. An inverse correlation of the gene expression between *ESR1* and *PAPSS1* was revealed in ROC plotter datasets of ovarian cancer. We also observed an inverse correlation between PAPSS1 and ERα in EOC and that the combination of low PAPSS1 and high ERα expression was associated with a survival benefit in EOC. ER pathway activity is consistent with the previous finding that the regulation of DNA repair activity is strongly associated with outcomes and response to chemotherapy in EOC [48]. However, the results here provide an alternative explanation by establishing a molecular connection between PAPSS1-mediated sulfation, ERα signaling and DNA repair. Our novel finding that the reduction in PAPSS1-mediated sulfation is indirectly responsible for the impairment of DNA repair mechanisms up-regulates ERα activity and estrogen-responsive gene expression, leaving PAPSS1-silencing EOC cells more sensitive to stimulation by cisplatin.

Hormonal ERa-targeted therapy, such as tamoxifen, fulvestrant, and aromatase inhibitors, prevents disease recurrence and reduces mortality from ERa-positive breast cancer. However, the positive response to ERa-targeted therapy in ovarian cancer is limited [49–51]. Traditionally, due to the estrogen etiology of ovarian cancer, estrogen replacement is not comprehensively recommended for most patients [52]. On the contrary, hormone replacement therapy (HRT) benefits the survival of EOC patients who have undergone surgical treatment [53, 54]. So far, the role of estrogen in EOC is still debated. Our results confirmed that EOC cells had higher ERα and estrogen-responsive gene expression by reducing the expression of PAPSS1 can sensitize tumors to cisplatin. Based on the present findings and previous reports, we hypothesized that ERα might be a PAPSS1-binding partner in EOC cells. Therefore, there is documented interplay between PAPSS1 and ER-signaling in tumorigenesis that may account for cisplatin resistance but also may be exploited for therapeutic development.

Despite our important findings, the present study has several limitations. First, this analysis was limited to two selected EOC cells and one basic anticancer drug. The exact mechanism through which PAPSS1 enhances the activity of the other cytotoxic agents, molecularly targeted drugs, and cancer immunotherapy drugs need to be further explored. Further studies on the precise molecular mechanisms involved would be required to explore this possibility.

## Conclusion

From a therapeutic perspective, our study first identified the potential of a combinational therapy using platinum drugs and PAPSS1 inhibitors to treat ovarian cancer patients. Our analysis also indicated for the first time a putative molecular role of the PAPSS1-ERa pathway as a basis for a better understanding HRT in EOC. These results may have an impact in the future on clinic prognosis and treatment of EOC patients.

## Methods

### Patients and tissue specimens

In total, 75 specimens of EOC and 31 normal ovarian epithelium tissues from benign tumor patients were analyzed in this study. EOC patients were 36–72 years old (mean age, 49.72 years); 18 cases were stage I-II, and 57 were stage III-IV. Histopathology and tumor grade were determined via pathology. None of the patients had been subjected to chemotherapy or radiotherapy before surgery, and all were treated with systemic platinum-based chemotherapy following surgery with a median follow-up period of 45 months. For experiments on platinum-based chemotherapy resistance, patients with EOC were divided into two groups (platinum-resistant and platinum-sensitive) according to the criteria described below. Patient response to chemotherapy was mainly evaluated according to National Comprehensive Cancer Network guidelines (version 1.2017, ovarian cancer) [55]: ‘sensitive’ vs. ‘resistant’ disease at 6 months.

Ovarian cancer tissue microarray (HOvaC160Su01) was obtained from Outdo Biotech Co Ltd (Shanghai, People’s Republic of China). Clinicopathological factors, such as age, FIGO stage, histologic grade, tumor size, lymph node metastasis, recurrence and platinum resistance, were collected from the database (http://www.superchip.com.cn/biology/tissue.html).

This study was approved by the Medical Ethics Committee of the First Affiliated Hospital of Anhui Medical University, and informed consent was obtained from 2017 to 2019.

### Sampling

All patient tissues were snap-frozen in liquid nitrogen within 30 min after resection and stored at -80°C. Frozen sections were then analyzed by RT-PCR analysis and immunohistochemistry (IHC).

### Reagents

Cisplatin was purchased from Hansoh Pharmaceutical Co. Ltd (Lianyungang, China). E_2_ was obtained from Sigma-Aldrich (St. Louis, MO, USA).

### Immunohistochemistry (IHC)

Tissues with a tumor cell ratio > 60% were directly included in the study. Formalin-fixed, paraffin-embedded OC samples and animal tissue was freshly cut. The sections (4 μm) were then incubated with polyclonal PAPSS1 antibody (1:1500, Abcam) and monoclonal ERα antibody (1:1000, Santa Cruz).

For sections of data analysis, an intensity score represented the average intensity of the positive cells: 0 (none); 1 (weak); 2 (intermediate); and 3 (strong). The proportion and intensity scores were then multiplied to obtain a total score ranging from 0 to 12.

### Cell lines and cell culture

Human chemoresistant ovarian cancer cells SKOV3 and chemosensitive ovarian cancer cells A2780 were purchased from Shanghai Huiying Biological Co. Ltd. Human ovarian surface epithelial cells (HOSEpic) were obtained from iCell Bioscience Inc (Shanghai, China). HOSEpic and A2780 were cultured in complete Roswell Park Memorial Institute (RPMI) 1640 medium (Hyclone, USA) supplemented with 10% newborn calf serum (NBCS, Gibco), while SKOV3 were cultured in McCoy’s 5A medium (BI, Israel) supplemented with 10% fetal bovine serum (FBS, Gibco) at 37°C in a humidified atmosphere containing 5% CO2.

### SiRNA transfections

Small interfering RNA (siRNA) against the *PAPSS1* gene was synthesized by GenePharma (Shanghai, China). The non-targeting si-NC was used as a negative control. Each siRNA was transfected into cells using Lipofectamine RNAiMAX (Thermo Fisher Scientific, USA) according to the manufacturer’s instructions and then incubated for 48 h. Cell transfection efficiency was verified by qRT-PCR and Western blot.

### Cell counting kit-8 (CCK-8) assay

Cells were seeded into 96-well plates at a concentration of 1 × 10^4^ cells with 100 μl of medium per well; 5 replicate wells were set at the same time. After cellular adhesion, cells were exposed to a gradually increased concentration (0, 5, 10, 15 and 20 µM, were used for A2780 cell adherence; 0, 50, 100, 150 and 200 µM were used for SKOV3 cell adherence) of cisplatin for 24 h. Then, 10μL of a sterile CCK-8 (Beyotime Biotechnology, China) was added to each well and incubated for another 4 h at 37 °C. The absorbance at 570 nm was determined using a microplate reader (Thermo, USA).

### Colony formation assays

Cells were seeded into 6-well plates at a concentration of 1 × 10^3^ cells per well. After incubation for 14 days, the colonies were fixed with 4% formaldehyde for 15 min, stained with 1% crystal violet for 30 min, washed with distilled water, dried overnight, and counted the next day. Clonal formation rate (CFR) was calculated using the following formula: *number of colonies containing* > *50 cells(CFR* = *[(no. of colonies formed/no. of cells seeded)* × *100%]*).

### Flow cytometry

A2780 and SKOV3 cells transfected with si-NC and siPAPSS1 were harvested for 48 h. Apoptosis was induced by cisplatin (2 or 50 μM) for 24 h. The cells were harvested in trypsin and washed twice with cold phosphate-buffered saline (PBS). After centrifugation, the cells were stained using the annexin V-FITC/propidium iodide Apoptosis Detection Kit (KeyGen BioTECH, Nanjing, China), following the manufacturer's instruction. Apoptotic cells were uncovered using flow cytometry (BD Bioscience, USA). For the cell cycle analysis, cells were single-stained with PI with the BD Cycle test plus DNA reagent Kit (BD Biosciences,USA). Data were analyzed using Cell Quest software (BD Biosciences, USA).

### Hochest 33342 stainnig

Cells were seeded into a 6-well plate sat a concentration of 1 × 10^5^ cells per well. Then, 24 h after cisplatin treatment, cells were fixed, washed twice with PBS and stained with Hoechst 33342 according to the manufacturer’s instructions (Beyotime Biotechnology, China). Cells were observed under a fluorescence microscope (Olympus, Japan).

### Quantitative real-time PCR

Total RNA was extracted from the tissues and cells using TRIzol reagent (Invitrogen, USA) according to the manufacturer’s protocol. The RNA was then subjected to reverse transcription to synthesize complementary DNA (cDNA) using the PrimeScript RT reagent Kit (TaKaRa, Japan). Quantitative real-time PCR was performed using the SYBR Green PCR master mix (TaKaRa, Japan) on the Light Cycler 96 Real-time System (Roche, Switzerland). The following primers were used: PAPSS1, BRCA1, BRCA2, MRP1, MRP2, CCND1, ESR1, and β-actin.The messenger RNA (mRNA) levels were calculated using 2^−ΔΔCT^ and normalized to *β-actin* mRNA levels.

### Immunofluorescence

The immunofluorescence was performed on the fixed cells grown on the round glass coverslips (Thermo Fisher Scientific, USA) in 35 mm cell culture dishes. The cells were incubated with primary antibody against H2AX (Abcam, ab195188,1:50) overnight at 4 °C, followed by rhodamine-conjugated anti-mouse secondary antibodies incubation for 1 h, and DAPI (Beyotime Biotechnology, China) as a nuclear stain. The cells were then examined under confocal fluorescence imaging microscope (TCSSP5; Leica, Mannheim, Germany).

### Western blotting

Total cell lines and tissues were harvested with ice-cold PBS and lysed in a lysis buffer containing a protease inhibitor cocktail. The proteins were quantified using BCATM Protein Assay Kit (Pierce, Appleton, USA). The Western blotting was performed according to the standard protocol. The primary antibodies used were: PAPSS1, ERα, CCND1, p-AKT, Bax, Bcl-2, BRCA1, BRCA2, MRP1, MRP2 and GAPDH. The images were detected with the enhanced chemiluminescence system (Tanon, China) and analyzed with a digital imaging system (Tanon).

### Lentiviral transfection

LV3 lentiviral siRNA particles targeting human PAPSS1 (target sequences: GTCTGGACATGCTTCCTAA,ACAAGTTTCATATCACCTT, and GATCGATTCTGAATATGAA) were obtained from GenePharma (Shanghai, China). A2780 and SKOV3 cells were transduced using the LV3 lentiviral siRNA starter kit (GenePharma) following the manufacturer’s instructions, and then selected with 1.5 µg/mL puromycin for 14 days. The clone that was isolated, propagated, and eventually used forthe murine xenograft study was derived from transduction with the PAPSS1-target sequence CCCAGUGCACAAUGGACAUTTAUGUCCAUUGUGCACUGGGTT. A non-silencing control cell line (shSCR) was generated in parallel with the PAPSS1-silenced cells. The shRNA-modified cells were used for the murine xenograft studies described below.

### In vivo chemosensitivity assay

Female 5-week-old athymic BALB/c mice were purchased from Bioray Laboratories Inc., Shanghai, China. All the animals were housed in a specific pathogen-free environment with a temperature of 22 ± 1 ºC, relative humidity of 50 ± 1%, and a light/dark cycle of 12/12 h. All animal studies (including the mice euthanasia procedure) were done in compliance with the regulations and guidelines of Huazhong Agricultural University institutional animal care and conducted according to the AAALAC and the IACUC guidelines.

A2780 and SKOV3 cells, stably transfected with shSCR and sh-PAPSS1. A2780 and SKOV3 cells were suspended in PBS (5 × 10^6^ cells/mL) and subcutaneously injected into the upper flank of nude mice (150 μl/mouse). Once the tumor reached a mean volume 25 mm, mice were randomly divided into 6 groups (5 mice per group): PBS, DDP, shSCR + PBS, shSCR + DDP, sh-PAPSS1 + PBS and sh-PAPSS1 + DDP. PBS (0.1 ml) or DDP (0.3 or 3 mg/kg) were peritoneally injected into the mice at 4-day intervals, respectively.

Tumor volumes were examined once a week. Seven weeks after modeling, mice were euthanized, and the primary tumors were excised, paraffin-embedded, formalin-fixed, and subjected to H&E and immunohistochemical (IHC) staining analysis for PAPSS1 (1:1500, Abcam) and Ki67 (1:500, Thermo Fisher) protein expression.

### ELISA for measurements of E_2_

Twenty-four hours post-transfection, the A2780 and SKOV3 cells were treated with cisplatin (2 or 50 μM) for 24 h, respectively. The culture supernatants were harvested, and the concentrations of E_2_ were measured using ELISA kits (RayBiotech,USA) according to the manufacturer's instructions. The dates were measured at 450 nm by an enzyme-linked immunosorbent assay plate reader (Model 680, Bio-Rad, Hercules, CA, USA). Experiments were performed three times independently.

### Luciferase assay

Cells were plated at a density of 10 × 10^4^ per well in 6-well plates 24 h before transfection. Each well was transfected with 0.4 μg of ERE-luciferase plasmid using Lipofectamine-2000 transfection reagent (Invitrogen) according to the manufacturer’s instructions. At 24 h posttransfection, the cells were treated with vehicle, E_2_ (1 nM), cisplatin (2 or 50 μM), and E_2_ (1 nM) + cisplatin (2 or 50 μM) for 24 h. Cell lysates were harvested 24 h later using MPER extraction reagent (Pierce), and luciferase assays were performed using the dual luciferase assay kit (Promega) according tothe manufacturer’s instructions. The luciferase activities were normalized to Renilla luciferase activity.

### Online database analysis

The online website GEPIA (http://gepia.cancer-pku.cn/) was used to analyze the PAPSS1 and ESR1 expression in ovarian cancer tissues and normal ovarian tissues. The TNMplot (https://tnmplot.com/analysis/) was used to employ the expression of PAPSS1 in tumor, normal and metastatic tissues of the ovary. The prognostic value of PAPSS1 and ESR1 expression, PFS and OS for treated with platinum-based chemotherapy were performed using the Kaplan–Meier Plotter platform (http://kmplot.com/). ROC plotter (http://www.rocplot.org), was used to detect PAPSS1 and ESR1 expression under different platinum responsiveness.

### Statistical analysis

For all quantitative analyses, data were analyzed with the SPSS version 21.0 (SPSS, Chicago, IL, United States) and expressed as the means ± SEM. The statistical comparison was carried out with independent samples T set. Spearman chi-square test was used to analyze the relationship between PAPSS1 expression and clinicopathological characteristics. In addition, Kaplan–Meier analysis was performed to assess the differences in survival rates. Each test was two-sided, and *P* < 0.05 was considered statistically significant.

### Supplementary Information

**Additional file 1:** **Table S1.** Primers used in the present investigation. **Table S2.** List of antibodies used in Western blot (WB), immunofluorescence (IF), And immunohistochemistry (IHC).

## Data Availability

All data generated or analyzed during this study are included in this published article.

## Abbreviations

EOC: Epithelial Ovarian cancer
PAPSS1: 3’-Phospoadenosine 5’-phosphosulfate synthase 1
PAPSS2: 3’-Phospoadenosine 5’-phosphosulfate synthase 2
HRR: Homologous combination repair
E_2_: Estradiol
ER: Estrogen receptor
Era: Estrogen receptor alpha
OS: Over survival
HRT: Hormone replacement therapy
IHC: Immunohistochemistry
CCK-8: Cell counting kit-8
