# Supplementary material for: The PAPSS1 gene is a modulator of response to cisplatin by regulating estrogen receptor alpha signaling activity in ovarian cancer cells
Source: J Ovarian Res. 2023 Sep 8;16:187. doi: 10.1186/s13048-023-01262-7 (PMC10486135; doi:10.1186/s13048-023-01262-7)
Supplement: Supplementary file 1 — Additional file 1: Table S1. Primers used in the present investigation. Table S2. List of antibodies used in Western blot (WB), immunofluorescence (IF), And immunohistochemistry (IHC). [file 13048_2023_1262_MOESM1_ESM.docx]

**Supplementary Tables**

**Supplementary table S1primers used in the present investigation**

| Target | Primer sequence (5’-3’) |
| --- | --- |
| PAPSS1 | F: CTTTGCATTTCAACTACGCAAC |
|  | R:CATCAAAGGAACATCGTCATCC |
| BRCA1 | F: CTCGCTGAGACTTCCTGGAC  R: TCAACTCCAGACAGATGGGAC |
| BRCA2 | F: TCGTGCTTTGCAAGATGGTG  R: TGTTCAGCAGATTCCATGGC |
| MRP1 | F:TGCTCACTTTCTGGCTGGTA  R:ACAGGACAAGACGA GCTGAA |
| MRP2 | F:TAGGTGCGTGGACTCTGGACATG  R:ACCTGGCAAATTACCCTTCAGAGC |
| CCND1 | F: ATTTCGGGCTTTTATTTGGATCGAT |
|  | R: TTCGGGAGGTGCTGGAATGC |
| ESR1 | F: TACTGCATCAGATCCAAGGGAA |
|  | R: CCTCGGGGTAGTTGTACAC |
| β-actin | F:GTGGCCGAGGACTTTGATTG |
|  | R: CCTGTAACAACGCATCTCATATT |
|  |  |

**Supplementary table S2 List of antibodies used in western blot (WB),**

**immunofluorescence (IF) and immunohistochemistry (IHC).**

**Antibodies used in the present investigation**

| **Target** | **Dilution in 5% milk in TBST** | | **Cat. Number** | **Supplier** | |  |
| --- | --- | --- | --- | --- | --- | --- |
| \| PAPSS1  ERa  CCND1  p-AKT(Ser473)  Bax  Bcl-2   \| BRCA1 (D-9)  MRP1  Ki67 (SP6)  GAPDH \| \| --- \| \| 1:1000  1:1000  1:500  1:1000  1:1000  1:3000  1:200  1:1000  1:500  1:2000 \| \| --- \| --- \| --- \| | | ab155600  sc-8002  ab185241  ab81283  #2722  #1017-1  sc-6954  #14685S  Thermo Fisher  TA-08 | | | abcam  Santa Cruz  abcam  abcam  CST  Epitomics  Santa Cruz  CST  MA5-14520  Zhongshanjinqiao | |
